# The Microbiome-TIME Axis: A Host of Possibilities

**DOI:** 10.3390/microorganisms11020288

**Published:** 2023-01-22

**Authors:** Tyler Joel Ross, Jun Zhang

**Affiliations:** 1 School of Medicine, University of Kansas, Kansas City, KS 66160, USA; 2 Department of Cancer Biology, University of Kansas Comprehensive Cancer Center, University of Kansas Medical Center, Kansas City, KS 66160, USA; 3 Division of Medical Oncology, Department of Internal Medicine, University of Kansas Comprehensive Cancer Center, University of Kansas Medical Center, Kansas City, KS 66160, USA

**Keywords:** microbiome, TIME, immunotherapy, cancer

## Abstract

Cancer continues to be a significant source of mortality and morbidity worldwide despite progress in cancer prevention, early detection, and treatment. Fortunately, immunotherapy has been a breakthrough in the treatment of many cancers. However, the response to immunotherapy treatment and the experience of associated side effects varies significantly between patients. Recently, attention has been given to understanding the role of the tumor immune microenvironment (TIME) in the development, progression, and treatment response of cancer. A new understanding of the role of the microbiota in the modulation of the TIME has further complicated the story but also unlocked a new area of adjuvant therapeutic research. The complex balance of tumor-permissive and tumor-suppressive immune environments requires further elucidation in order to be harnessed as a therapeutic target. Because both the TIME and the microbiome show importance in these areas, we propose here the concept of the “microbiome-TIME axis” to review the current field of research and future directions.

## 1. Introduction

The microbiome represents the sum of all bacteria, viruses, archaea, fungi, algae, and their genomes within the host [1]. It has been found that these microbes have a profound effect on a variety of elements of human development and physical health [2]. The microbiome plays an important role in the maturation and education of the host immune system [3]. The effects of the microbiota on the course of cancer and response to therapy are mediated primarily via modification of the immune system [4]. While the immune system is well established as a first-line defense against cancer, its role is complicated by the spatiotemporal interactions with the tumor. The tumor immune microenvironment (TIME) describes the complex balance and interaction of tumor cells, immune cells, and supporting cells within a tumor [5]. These cell types include lymphocytes, macrophages, dendritic cells, neutrophils, and fibroblasts. The interactions between these cells, the tumor, and the microbiota are mediated by direct cellular interactions, chemokines, cytokines, and cellular metabolites as we will discuss in detail below. The communication between tumor cells and the surrounding immune cells can result in the reprogramming of the immune cells within the TIME thus creating an immunosuppressive environment that favors cancer progression [6]. The degree to which this reprogramming takes place can be affected by the patient’s microbiota which serves as an integral component and modulator of the TIME [7], hence we name it the “microbiome-TIME axis”. The microbiome-TIME axis refers to the unique pathways of communication between the host microbiota and the TIME which includes the tumor cells, immune cells, signaling molecules, and metabolites. As more research is conducted on the role of microorganisms within and remote to the TIME, it is apparent that the microbiome is involved in many, if not all, of the hallmarks of cancer. The following review summarizes the current research regarding the role of microbiota in the modulation of each category of immune cells within the TIME and proposes future directions for research regarding the modulation of the microbiome-TIME axis.

## 2. Significance of TIME

Clinically, the characterization of the TIME has shown an association with the prognosis and response to the treatment of patients with tumors [8]. Modulation of the TIME to reverse the immunosuppressive effects appears to be a promising therapeutic option. With the approval of new immunotherapies targeting immune checkpoints on tumors and immune cells, the importance of categorizing the TIME has become more evident. The analysis of the TIME has significant implications in the treatment of cancer as it can predict the response to immune checkpoint inhibitors [9]. As a result, testing for molecular markers such as microsatellite instability (MSI) and programmed cell death protein/ligand 1 (PD-1/PD-L1) has become the standard of care since these findings can help guide personalized immunotherapy treatment [10]. Additionally, recent studies have correlated the composition and diversity of the microbiome to both the patient’s response to immunotherapy and the experience of adverse events associated with treatment [11,12,13,14]. As such, new interest has been raised in characterizing and modulating a patient’s microbiome as an opportunity for further optimization of the TIME via alterations in the microbiome-TIME axis.

## 3. Composition of TIME and Its Potential Impact by Microbiota

Despite the variation in the TIME across individuals and cancer types, several general classifications of TIME have been presented. These generalized descriptions do not represent all of the possible tumor immune microenvironments. Further characterization of TIME is needed to develop more targeted approaches to cancer therapy. The individual classification of a patient’s TIME represents a potential approach to personalized precision medicine.

Two general classifications of TIME have been well documented across tumor types: infiltrated-excluded (I-E) and infiltrated-inflamed (I-I) [15]. The antitumor effects of each of these environments depend on the spatial distribution and immune composition of the tumor [15]. Infiltrated-excluded tumors are characterized by the presence of cytotoxic T lymphocytes (CTLs) at the periphery of the tumor without invasion into the tumor core [15]. The immune cell exclusion can be either physical due to fibrosis [15] or chemical due to alterations in chemokine expression within the tumor [16]. Regardless of the process, immune cell exclusion results in an immunologically “cold” tumor and suboptimal immune response [16]. The relatively impaired immune cell penetration into the tumor significantly impairs the efficacy of immunotherapy since many of these therapies rely on the physical interaction between CTLs and cancer cells. The vast number of physical and chemical processes that mitigate immune cell exclusion in the TIME all represent potential targets of adjuvant therapy.

Alternatively, infiltrated-inflamed tumors show CTLs throughout the tumor and are associated with increased levels of IFN-γ [15]. The greater immune cell penetration into the tumor allows for a more robust immune response in cancer and an overall improved response to immunotherapy. Despite a greater concentration of infiltrating CD8^+^ T cells, the presence of other immunosuppressive members of the TIME, such as regulatory T cells (Tregs), results in the cells being dysfunctional and having suboptimal cancer cell killing potential [17]. Despite the binary delineation, the TIME of each patient exists along a spectrum of these descriptions.

**Figure 1:**
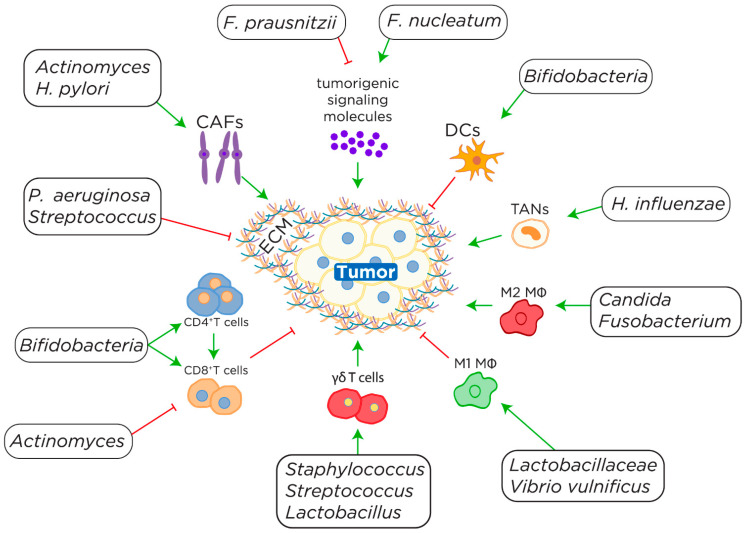
The regulatory effects of specific microbiota on the TIME. Microbiota have a variety of inducing (green arrows) and inhibitory (red arrows) effects on immune cells. These effects are mediated through the modulation of immune and supportive cells within the TIME and translate into tumor-suppressive (red arrows) and tumor-promoting (green arrows) effects. Dendritic cells (DCs), M1 macrophages (MΦ), and CD8^+^ T cells generally have an antitumor response within the TIME. On the other hand, cancer-associated fibroblasts (CAFs), M2 MΦs, tumor associated neutrophils (TANs), and γδ T cells promote tumorigenesis. It is possible that the same microbiota may have different effects on the TIME depending on the context.

The TIME is further complicated by the interactions with the intratumoral and host microbiota. The composition of the microbiome determines the overall balance of inflammation [18]. The species-specific colonization of the microbiome and tumor can have both positive and negative effects on tumorigenesis and the antitumor response via direct effects on cancer and immune cells. The TIME extends beyond the immediate tumor region and is impacted by the microbiome through a myriad of pathways (i.e., via the microbiome-TIME axis). These pathways include modulation of the extracellular matrix [19], changes in cancer-associated fibroblasts [20], altered efficacy of innate and adaptive immune cells [21,22], and fluctuations in signaling molecules [23] (Figure 1). Since the immune system and the microbiota are in constant and dynamic interaction, the microbiota plays an integral role in shaping the immune system and thus the TIME. Therefore, it is logical that aberrations in the microbiome can have an effect on the clinical course of a tumor as well as the response to therapy. The importance of the microbiome in the response to immunotherapy has been well documented in a variety of cancer types including metastatic melanoma, non-small cell lung cancer, renal cell carcinoma, and urothelial cancers [13,24,25]. It is postulated that the differences in immunotherapy response are at least partially due to the microbiome’s role in altering both the TIME and relevant immune signaling pathways as discussed below.

### 3.1. Extracellular Matrix

The extracellular matrix (ECM) connects all components of the TIME, acting like a scaffold; therefore, its composition affects the TIME. The ECM is composed of a variety of compounds including collagen, fibronectin, and laminin [26], and generally provides mechanical support, growth factors, and cytokine-binding ability [27]. Tumors with well-organized collagen and a low-volume ECM have poorer responses to therapy due to poorer drug penetrance within the tumor [27]. High collagen ECMs have been shown to have less intratumoral vasculature and are associated with worse chemotherapy delivery to the tumor in pancreatic ductal adenocarcinoma [28]. The organization and composition of the ECM also contribute to the ability of immune cells to invade the tumor. Proteoglycans found within the ECM, such as versican, regulate immune cell infiltration into the tumor via cell surface receptors [29]. While previously thought to be sterile sites, bacteria of the microbiome can invade tumors to directly alter the ECM. While the mechanism is unclear, it is postulated that the hypoxic nature of the tumor’s core as it grows provides optimal conditions for the growth of anaerobic bacteria [30]. Thus, it is possible that at least local microbiota can migrate to the site of the tumor. The variation in the physical tumor environment with regard to oxygenation levels allows selective colonization by bacteria [30]. Tumor hypoxia also is a selective pressure for angiogenesis [31] which provides another route for bacteria to possibly colonize the tumor via hematogenous spread. Streptokinase from *Streptococcus pyogenes* has anti-angiogenic effects which can limit tumor cell invasion and metastasis [32]. Streptokinase has also been shown to improve treatment response to chemotherapy agents in colon cancer [33]. Bacterial enzymes, such as alkaline protease and elastase, degrade ECM and facilitate bacterial invasion of the tumor [19]. Additionally, the alkaline protease and elastase of *P. aeruginosa* have been found to impair the antitumor immune response by inhibiting IL-2 proliferation of lymphocytes and degrading IFN-γ (Figure 1) [34]. The immunosuppressive role of these enzymes potentiates their impairment of the antitumor response. Streptococcal pyrogenic exotoxin B cysteine protease cleaves transmembrane proteins weakening the epithelial barrier and allowing for bacterial penetration of the ECM and further modulation of the TIME [19]. *Streptococcus* abundance has been shown to be increased in the urine of urothelial carcinoma patients [35] suggesting that it might play a role in the modification of the TIME. The degradation of the ECM by bacteria or fungi provides a potential environment for tumor metastasis [19]. Therefore, the microbiota have an impact on the TIME via the modulation of the ECM.

### 3.2. Cancer-Associated Fibroblasts

Cancer-associated fibroblasts (CAFs) are a major component of the TIME and are closely associated with the ECM. CAFs are a very heterogeneous cell group that undergoes expansion following the tissue dysfunction accompanying cancer [36]. Recently, CAFs have gained attention as a potential therapeutic target because of their important role in modulating the structural and immunological components of the TIME [20]. CAFs alter cancer progression by modulating the physical ECM environment and by secreting immunomodulating cytokines which impact the infiltration of immune cells into the TIME [37,38].

The effects of CAFs on the TIME are further complicated by microbiota. Recently, colonization with *Actinomyces* species has been associated with young-onset colorectal cancer [39]. *Actinomyces* have been shown to co-localize with and preferentially infect CAFs and impair CD8^+^ T cell infiltration by toll-like receptor 2 (TLR2) signaling (Figure 1) [39]. In mouse models, infection with *H. pylori* was found to differentiate fibroblasts into cells that possess CAF characteristics and secrete pro-inflammatory cytokines such as IL-6 (Figure 1) [40]. Infection of CAFs with *H. pylori* results in the upregulation of vascular adhesion molecule 1 (VCAM1) via JAK/STAT signaling [41]. Upregulation of VCAM1 is associated with tumor progression and lymph node metastasis and is an overall negative prognostic indicator [41].

### 3.3. Innate Immune Cells

Innate immune cells are integral components of the microbiome-TIME axis and contribute to the antitumor response. Like in infection, innate immune cells serve as the body’s first recognition of and response to cancer. Macrophages and dendritic cells recognize cancer in response to damage-associated molecular patterns (DAMPs) such as damaged DNA [42]. Natural killer cells on the other hand respond by recognizing and killing tumor cells that do not express MHC molecules [43]. Macrophages, a type of innate immune cell, can be categorized into M1 and M2 macrophages. M1 macrophages are induced by IFN-γ, lipopolysaccharide (LPS), or lipoteichoic acid (LTA) typically in response to an infection [44,45]. M1 macrophages play an important role in responding to bacterial and viral infections by producing reactive oxygen species (ROS). Alternatively, M2 macrophages are activated by exposure to cytokines such as IL-4, 10, or 13 [44]. M2 macrophages are associated with wound healing and are generally thought to dampen the immune response. The effectiveness of these cells to contribute to the antitumor response is heavily dependent on the microbiome and its role in the differential activation of these cell subtypes. M2 macrophages play a role in angiogenesis and immune suppression thus favoring tumor growth [46]. In mouse models, dietary restriction of protein has been found to reverse the immunosuppressive role of tumor associated macrophages (TAMs) and inhibit tumor growth in prostate and kidney cancer [47]. The dietary protein restriction also resulted in a shift in the microbiome with an increase in *Lactobacillaceae* members and a decrease in *Prevotellaceae* and *Ruminococcaceae* members suggesting that the microbiome may contribute to the polarization of TAMs (Figure 1) [47]. *Vibrio vulnificus* flagellin B has also been shown to enhance the polarization of TAMs from M2-like to M1-like resulting in an enhanced antitumor response (Figure 1) [21]. Furthermore, *Bifidobacterium bifidum*-based hybrid bacteria have been shown to penetrate tumor tissue through hypoxia targeting to have direct contact with TAMs [48]. Colonization of the TIME by this hybrid bacteria resulted in the polarization of TAMs toward the M1 phenotype, and an enhanced immune effect was achieved (Figure 1) [48].

*Bifidobacteria* have been found to colonize the TIME and modulate the response to CD47 blockade [49]. *Bifidobacteria*’s enhancement of the antitumor response to CD47 blockade is mediated by STING signaling in dendritic cells (Figure 1) [49]. Dendritic cells exhibit a cross-priming ability that bridges the innate and adaptive immune systems. Without the colonization of the TIME with *Bifidobacteria,* the cross-priming did not occur which resulted in reduced efficacy of CD47 blockade treatment [49]. Rather than a binary of innate and adaptive immunity, it appears that the host microbiome is a necessary component for the cooperation of these two systems.

Neutrophils are another key player in the TIME and are implicated in the growth and metastasis of tumors. Neutrophils are recruited by tumoral secretion of chemokines such as IL-8, macrophage inflammatory protein-1α (MIP-1α), and human granulocyte chemotactic protein-2 (huGCP-2) [31]. Once recruited, tumor associated neutrophils (TAN) release tumor necrosis factor (TNF) which is associated with cancer metastasis to lymph nodes [31]. Like other innate immune cells, TANs are also influenced by the host’s microbiota. Using both animal models and patient lung cancer samples, it was found that *Haemophilus influenzae* induced IL-17C expression, which promoted neutrophilic inflammation in the tumor microenvironment (Figure 1) [50]. The neutrophilic inflammation driven by IL-17C resulted in enhanced tumor growth [50].

### 3.4. Adaptive Immune Cells

Adaptive immune cells, such as T cells, play important roles in the antitumor response. In particular, the cells work with immunotherapy to improve the antitumor response. The recruitment and efficacy of adaptive immune cells depend heavily upon the microbiome-TIME axis. Adaptive immune cells can be influenced by global and local alterations in signaling molecules. It has been reported that the dysbiosis of commensal microbiota in the lung, which mainly involves the genera *Staphylococcus*, *Streptococcus* and *Lactobacillus*, activates lung-resident γδ T cells (Figure 1) [51]. The γδ Τ cells secrete IL-17 and other pro-inflammatory cytokines to promote inflammation thereby stimulating lung adenocarcinoma tumor cell proliferation [51]. *E. coli* not only is capable of colonizing tumor tissues but it is also associated with the induction of CD8^+^ T cells which produce a significant antitumor response resulting in tumor regression in mouse models [22]. *Bifidobacteria* species have also been implicated in the modulation of the adaptive immune response. *Bifidobacterium breve* has been shown to express epitopes that have high homology to human melanoma antigen [52]. The similarity of these antigens allows for cross-reactivity, and *Bifidobacterium* colonization in the gut promotes T cell amplification [52]. Thus, bacterial colonization distal to the tumor has the potential to prime the immune system to attack the tumor via this antigenic cross-reactivity. This represents yet another example of the microbiome-TIME axis. Further, *Bifidobacteria* probiotic supplementation has been found to enhance the antitumor response via increased recruitment of CD8^+^ T cells in patients with colorectal cancer (Figure 1) [53]. *Bifidobacteria* probiotics also have been shown to regulate normal microbial flora to prevent dysbiosis and colonization with potentially tumor supportive species [53]. Alternatively, gastric tumors colonized with *Methylobacterium* exhibit lower levels of CD8^+^ tissue-resident memory T cells (TRM) and an overall poorer prognosis [54]. High levels of TRM cells are associated with an improved response to immunotherapy [55]. Moreover, TRM cells play an important role in lasting protection against tumors [56]. These findings together suggest that modification of the gut microbiome has both local and distal effects on TIME via modulation of the adaptive immune system.

### 3.5. Signaling Molecules

All of the aforementioned cell types must migrate to the site of the tumor from global circulation in order to exert their effects. Therefore, cytokines, produced within the TIME play an important role in the recruitment, composition, and effectiveness of the resident immune cell population. The composition of the microbiota can result in changes in chemokine expression. For instance, *Faecalibacterium prausnitzii* has been found to produce metabolites that inhibit the NF-κB and CXCL8 pathways (Figure 1) [23]. NF-κB activation plays an integral role in tumorigenesis by increasing cell proliferation and enhancing metastasis [57]. Inhibition of NF-κB, as in the case of *F. prausnitzii*’s effects, has been shown to protect tissues from inflammation in the tumor microenvironment and inhibit tumorigenesis and metastasis [57]. CXCL8 has a wide range of negative implications including promoting tumor proliferation, invasion, angiogenesis, and resistance to anticancer therapy [58]. Cancer cells have been shown to secrete CXCL8 to maintain the aggressive phenotype and inhibit apoptosis [59]. In fact, CXCL8 is so pro-tumorigenic that it has been proposed as a prognostic marker in cancer patients [60]. Thus, inhibition of the CXCL8 pathway by *F. prausnitzii* metabolites is a notable TIME modulation.

Alternatively, *Fusobacterium nucleatum* has been shown to accelerate intestinal tumor growth via the recruitment of M2-like TAMs and myeloid-derived suppressor cells (MDSCs) as well as the upregulation of NF-κB (Figure 1) [61]. M2-like TAMs are associated with an immunosuppressive TIME that is favorable for cancer progression. *Streptococcus gallolyticus* colonization has been associated with increased IL-1 and IL-8 levels in colorectal cancer patients [62]. IL-1 is a pro-inflammatory cytokine and IL-8 is a potent angiogenic cytokine both of which play roles in tumorigenesis and progression. Angiogenesis is imperative for the growth of the tumor since it increases nutrient and oxygen delivery to the increasingly hypoxic tumor. Enterotoxigenic *Bacteroides fragilis* colonization results in high levels of IL-17 and IL-23 expression driving a T_H_17 inflammatory response [63]. T_H_17 cells have been found to preferentially infiltrate tumors and secrete pro-inflammatory cytokines which promote tumor growth and impair the antitumor immune response [64]. Figure 1 summarizes the general immunoregulatory pathways implicated by different microbiota species in the TIME.

## 4. Microbiome-TIME Axis in Tumorigenesis and Cancer Treatment

**Figure 2:**
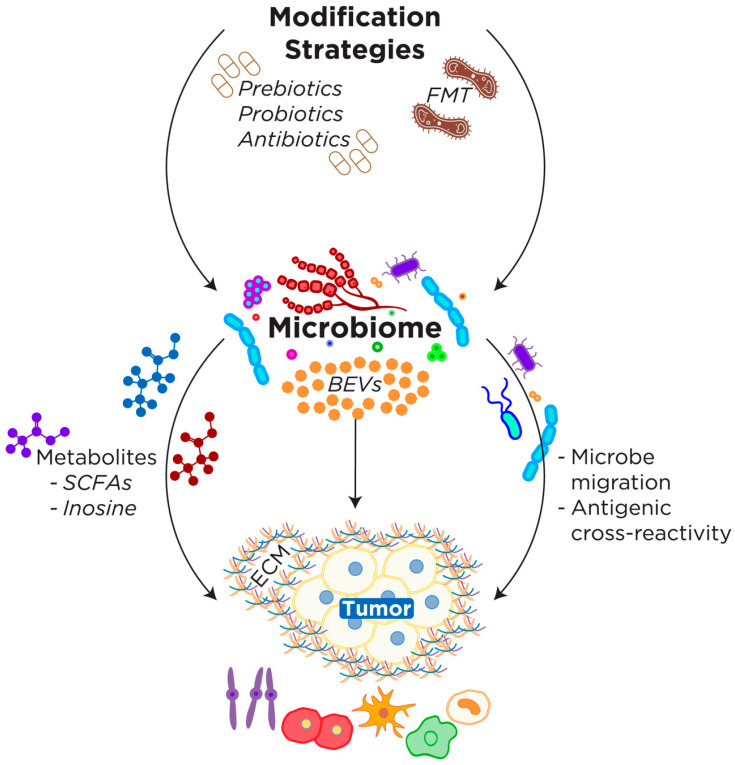
The Microbiome-TIME axis and associated modification strategies. The microbiome-TIME axis refers to the effect that microbiota have on the TIME through one or a combination of the following possible mechanisms including local (and possibly remote) microbe migration, antigenic cross-reactivity, metabolites and bacterial extracellular vesicles (BEVs). Microbes can directly migrate to and colonize the TIME, or they can produce metabolites, such as short chain fatty acids (SCFAs) that enter circulation to alter the TIME. Many modification strategies have been proposed to alter the patient’s microbiome and, thus, alter the TIME. These methods are discussed in greater detail below but include FMT, antibiotics and pre/probiotics.

The human microbiome consists of a diverse population of organisms with significant implications for human health. Interpersonal variation in the microbiome results in the presence of different metabolites, cytokines, and other immunomodulating pathways of the microbiome-TIME axis that influence oncogenesis and treatment response (Figure 2). In fact, in mouse models, disruption of the gut microbiome impacted the response of tumors to immunotherapy [65]. Studies in humans have also found an association between certain bacterial species within the microbiome and the patient’s response to immunotherapy [24,25,66]. Intestinal microbiota have previously been implicated in the modulation of cancer immunotherapy [65] and chemotherapy [67]. For instance, mice treated with antibiotics exhibited worse overall survival and poorer response to oxaliplatin as compared to control mice [67]. Additionally, dysbiosis induced by antibiotics promoted the growth of hepatocellular carcinoma (HCC) [68]. Mechanistically, this occurred by increasing levels of IL-25 inducing the selective differentiation of M2 macrophages which promoted a tumor-permissive immune environment [68]. The antibiotic-induced dysbiosis also caused colonic hyperplasia of epithelial tuft cells which, in turn, secreted more IL-25 further perpetuating this effect [68]. The gut microbiome has been associated with patients’ response to PD-L1 immunotherapy with high levels of *Bifidobacteria* improving treatment response [69]. This suggests that the gut microbiome may explain the disparities in treatment response among patients who respond to immunotherapy and those who do not [25].

Bacteria have conflicting impacts on tumorigenesis depending on the species, immune response, and location. Many commensal bacteria have a protective effect against cancer. On the other hand, some bacterial infections activate phagocytic immune cells such as macrophages which produce ROS causing potentially DNA-damaging oxidative stress on the surrounding cells [30]. Chronic dysbiosis can lead to chronic inflammation which promotes carcinogenesis via the activation of NF-κB [30]. More recently, specific bacteria have been isolated from solid tumors that vary depending on the cancer type. This suggests that certain bacteria may uniquely play a role in the oncogenesis of specific tumor cells [67]. Some microbes have been directly implicated in the oncogenesis of tumors such as *Helicobacter pylori*’s ability to cause gastric cancer via the inhibition of the p53 tumor suppressor protein [70]. Additionally, *F. nucleatum* has been implicated in the tumorigenesis of colorectal cancer via its effects on NF-κB signaling and immunosuppressive effects [61]. A summary of the microbiota and immunological factors that influence the development of various cancer types is shown below in Table 1 with demonstrated mechanisms. Several microbes were reported to create a tumor-permissive environment via their impact on inflammation and immunosuppression. More causal links will likely be identified as the role of the microbiome-TIME axis is more closely studied.

### 4.1. Microbial Metabolites

Other than the aforementioned impacts on the microbiome-TIME axis, such as the migration of local microbiota and antigenic cross-reactivity, microbiota may create molecules through metabolic pathways that benefit cancer cells and further tumorigenesis. *Clostridium* and *Eubacterium*, members of the *Firmicutes* family, for instance, have been found to convert primary bile acids into secondary bile acids which can damage DNA, a hallmark of oncogenesis and an opportunity for the cancer cells to gain additional mutations [71]. Alternatively, many metabolites of the microbiota have antitumorigenic immunomodulatory roles which are discussed below.

#### 4.1.1. Short-Chain Fatty Acids

For example, short-chain fatty acids (SCFAs) produced by bacteria have been found to play a role in the differentiation of effector CD8^+^ T cells to functional memory T cells [72]. Clinically, patients with high levels of fecal SCFAs experience longer progression-free survival in solid-tumor cohorts receiving immunotherapy [73]. The proposed mechanism involves SCFAs increasing the expression of IFN-γ in CD8^+^ CTLs and IL-17-secreting CD8^+^ T cells resulting in an enhanced antitumor response [73]. Production of SCFAs from intestinal microbiota has also been shown to prevent breast cancer metastasis by acting on G protein-coupled receptors [7]. In particular, SCFAs have been shown to upregulate *CDH1* (E-cadherin) expression via the activation of FFAR2 receptors resulting in decreased tumor invasion and metastasis [74]. Butyrate, a SCFA, has been found to boost the antitumor response of CD8^+^ T cells by promoting IL-12 signaling pathways via an ID2-dependent pathway [75]. Butyrate has also been shown to inhibit the growth of cancerous colonocytes by inhibiting glucose transport and glycolysis [76]. This is of particular note since cancer cells preferentially uptake and utilize glucose for metabolism as per the Warburg effect. Metabolites, such as SCFAs, appear to have a direct tumor suppressive effect in addition to improving the antitumor immune response. The production of butyrate from gut microbes results in a global increase in serum butyrate allowing gut microbes to alter the distal TIME. This suggests that the effects of the microbiome on the TIME extend well beyond the local physical interactions within the tumor.

#### 4.1.2. Inosine

Similarly, *Bifidobacterium pseudolongum*, *Lactobacillus johnsonii*, and *Olsenella* species have been found to produce elevated levels of inosine, another bacteria-derived metabolite [77]. In various tumor models, inosine increased the response to checkpoint inhibitor immunotherapy by causing preferential CD4^+^ T_H_1 differentiation in the presence of a costimulatory signal from IFN-γ [77]. CD4^+^ T_H_1 cells play an important role in priming and inducing CD8^+^ T cells and are necessary for determining the magnitude of the cytotoxic response [78]. CD4^+^ T_H_1 cells acquire MHC complexes by dendritic cell (DC) activation allowing them to deliver targeted IL-2 to CD8^+^ T cells which prolongs activated CD8^+^ T cell survival and improves infiltration into the tumor [78]. Thus, CD4^+^ T cells play an important role in helping CD8^+^ T cells overcome the immunosuppressive barriers associated with the TIME. The context-dependent role of inosine further illustrates the complex interactions within the microbiome-TIME axis that modulate patients’ responses to immunotherapy.

### 4.2. Extracellular Vesicles

Extracellular vesicles (EVs), such as exosomes, are nanoscale vesicles that contain constituent molecules of the cells that produce them. EVs can include RNA, DNA, proteins, lipids, and metabolites. The molecular composition of EVs varies depending on cell type and is postulated to be a result of targeted endosomal sorting [79]. Since all cell types produce EVs, they represent a point of complex intercommunication between cancer and non-cancer cells [80]. Due to the diversity of their contents, EVs have been found to play a role in every step of oncogenesis including initiation, promotion, progression, and metastasis [80].

EVs derived from bacteria also modulate the TIME. Bacteria-derived EVs (BEVs) contain a wide variety of metabolites, proteins, and genetic material making them a unique player in the interdomain communication between bacteria and eukaryotes. Bacterial genetic material has been found to incorporate into the host genome at higher rates in cancer cells [81]. The uptake of BEVs has been well documented in gastrointestinal tumors where the tumor is in close proximity to the microbiome [82]. The DNA from these uptaken BEVs has been found to incorporate into the gastrointestinal tumor genome suggesting that bacterial DNA may play a role in oncogenesis [82]. In addition, BEVs are speculated to penetrate the gut epithelial lining, enter the bloodstream, and get into the TIME of remote tumor sites [82,83]. BEVs are detectable in patient plasma [84] and are at significantly higher levels in patients with a variety of disease states including cancer patients with therapy-induced intestinal mucositis [85]. The interaction between BEVs and host cells appears to be mediated in part by the interaction of BEV surface microbe-associated molecular patterns (MAMPs) with host cell pattern recognition receptors (PRR) [81]. BEVs that accumulate in tumor tissues have been found to induce the secretion of CXCL10 and IFN-γ resulting in the suppression of tumor growth and decreased metastasis [86]. The immunomodulatory effect of BEVs has been demonstrated in a wide variety of cell types including gut epithelial cells, endothelial cells, and immune cells [87]. BEVs modulate the function of immune cells via the interaction of MAMPs with TLRs to initiate PRR signaling to promote an immune response [48]. The mechanism for the uptake of BEVs into other cell types is still an area of ongoing research. The effect of BEVs is species-specific with *Pseudomonas aeruginosa* BEVs acting on epithelial cells to induce the production of CXCL8 while *Acinetobacter baumannii* induces IL-1 and IL-6 production to drive immune cell infiltration [87]. Since not all BEVs facilitate the same immunologic response, it is important for future projects to elucidate the impact of different bacterial species’ EVs on the antitumor immune response. A greater understanding of the effect of specific species’ EVs would open the door to harnessing their power as an adjuvant cancer therapy.

## 5. Clinical Implications and Future Directions of Microbiome-TIME Axis

Clinically, there is still much to learn from the microbiome-TIME axis and its implications on tumor growth and invasion, as well as its correlation to therapeutic responses. While much of the research regarding the TIME has been conducted in mouse models, the studies conducted in humans appear to yield supportive results. For instance, the dietary intake of patients has been linked to differences in the intratumoral bacterial species isolated from breast cancer patients compared to healthy patients [88]. This suggests that the TIME is dynamic and modifiable through the microbiome. As previously discussed, different species within the microbiome confer beneficial immunomodulation while other species result in a cancer-promoting environment. Because of the microbiome-TIME axis, it has been proposed that the microbiome could be used as a clinical predictor of disease course and to predict the likelihood of response and adverse events related to immunotherapy treatment [14,66]. It is imperative that further work is conducted to identify optimal combinations of bacteria so as to optimize the antitumor response and minimize adverse events.

Furthermore, it is necessary to identify and develop effective and non-invasive methods to alter the microbiome of patients in order to optimize the TIME and improve the response to cancer therapies. Current methods, such as fecal microbiota transplantation (FMT), carry many risks including infection and have varying efficacy rates [89]. In fact, the FDA issued a safety alert concerning the use of FMT because of the observed risks of the transplantation of infectious pathogens [90]. Despite these risks, FMT has shown efficacy in mouse models. FMT in germ-free mice from patients who responded to PD-1 blockade resulted in significantly higher numbers of CD8^+^ T cells within tumors when compared to tumors from mice who received FMT from non-responding patients [25]. The response was mediated by high levels of *Akkermansia muciniphilia* in the stool, and supplementation with bacteria of this strain yielded similar results following the non-responder FMT [25]. More importantly, FMT was also found to be an effective treatment in melanoma patients in a phase 1 study [91]. Table 2 contains a summary of the role of microbiota on immunotherapy response. A more comprehensive list and analysis were reported in our previous publication [13]. Currently, our understanding of the microbiome’s response to FMT and the subsequent alterations of the TIME remains inadequate and warrants further investigation before this is considered a widely used adjuvant therapy.

Because of the limited risk of infection, BEVs appear to be an attractive option for modifying the microbiome-TIME axis. Currently, BEVs are being studied as a potential drug delivery system since they can effectively traffic to the site of the tumor [94]. This targeted delivery technique would also limit side effects by decreasing the need for systemic administration of chemotherapy or immunotherapy drugs. Additional studies using *E. coli* EVs have shown positive results in improving the immune response in colon cancer and inducing tumor regression [86]. However, further investigation needs to be conducted to determine the appropriate method of administration and to confirm their safety. A further challenge to this approach is the feasibility of producing EVs on a large enough scale to be utilized as a therapeutic treatment. Further work must be conducted to understand the mechanism by which EVs migrate to the TIME.

Administration of probiotics, which contain live bacterial strains, is yet another approach to modifying the microbiome and, thus, the TIME. Many probiotics are readily available but developing a mixture of strains to effectively improve the therapeutic response is a target of future work. Additionally, it is a challenge to remodel the microbiome due to bacterial competition and the potential for dysbiosis.

Prebiotics have also been proposed as a way to modify the microbiome and TIME. Prebiotics are defined as substrates that are selectively utilized by host microorganisms and confer a health benefit [95]. Unlike probiotics which modify the microbiome by adding bacteria directly, prebiotics alter the microbiome by providing substrates that aid the growth of microbes that are already colonizing the patient’s microbiome. Prebiotic and dietary interventions have shown therapeutic potential [96]; however, it is more difficult to precisely tailor the changes in the microbiome using this approach since the effect depends on the bacteria that already exist within the patient’s microbiome. This approach is further complicated by the converse relationship between the microbiome and the host’s diet. The gut microbiome plays a role in food digestion and absorption, but the host diet also shapes the microbiome composition. For these reasons, the direct effects of prebiotic supplementation on the TIME need to be elucidated.

## 6. Conclusions

The aforementioned techniques hold promise as adjuvant therapies to immunotherapy treatment and have the potential to narrow the response disparities that currently exist among patients treated with immunotherapy. The microbiome has complex interactions with the TIME that are dependent on the diversity of the colonized species, the location of colonization, and the patient’s immune profile. The microbiome’s impact on the TIME can be thought of as a balance of tumor-permissive and tumor-restrictive contributions that in total contribute to the clinical course of the disease. Nevertheless, modification of the microbiome as a method to favorably alter the TIME is a field of research with a host of therapeutic possibilities. Moving forward, it is imperative that the patient’s microbiome-TIME axis is considered when developing, researching, and prescribing cancer therapeutics in order to achieve the best possible clinical outcomes.

## Figures and Tables

**Table 1:** A summary of the role of microbiota on the development and progression of cancer.

Microorganism	Target Cell	Mechanism
*P. aeruginosa* [34]	Lymphocytes	Inhibition of IL-2 signaling
*S. pyogenes* [32]	CAFs/endothelial cells	Production of an anti-angiogenic streptokinase
*H. pylori* [41,70]	CAF	Upregulation of VCAM1 resulting in increased metastasis
	Gastric cells	Inhibition of the p53 tumor suppressor
*F. nucleatum* [61]	M2-like macrophages	Upregulation of NF-κB
*B. fragilis* [63]	T_H_17 cells	Increased levels of IL-17 and IL-23
*Clostridium* [71]	Varying	DNA damage from the conversion of primary to secondary bile acids
*Eubacterium* [71]		

**Table 2:** The effects of gut microbiota on immunotherapy response.

Author	Microbe	Effect
Routy et al. [25]	*Akkermansia municiphilia*	Associated with an increased response to anti-PD-1 immunotherapy
Sivan et al. [69]	*Bifidobacterium*	Associated with an increased response to anti-PD-1 immunotherapy
Vetizou et al. [24]	*Bacteriodes*	Associated with an increased response to anti-CTLA-4 immunotherapy
Gopalakrishnan et al. [92]	*Faecalibacteria, Ruminococcaceae, Clostridiales*	More abundant in patients who respond to anti-PD-1 immunotherapy
Dubin et al. [93]	*Bacteriodes*	Lowered risk of anti-CTLA-4 treatment-associated colitis
Chau et al. [14]	*Bifidobacterium*	High abundance correlated to lower immune-mediated toxicity from chemoimmunotherapy
	*Clostridiales*	High abundance in responders to chemoimmunotherapy

## Data Availability

The data presented in this study are available in the references cited. For additional information if applicable, please contact the corresponding author.
